# Interradicular distance and alveolar bone thickness for miniscrew insertion: a CBCT study of Persian adults with different sagittal skeletal patterns

**DOI:** 10.1186/s12903-021-01891-8

**Published:** 2021-10-17

**Authors:** Amin Golshah, Mahya Salahshour, Nafiseh Nikkerdar

**Affiliations:** 1 Department of Orthodontic, School of Dentistry, Kermanshah University of Medical Sciences, Shariati Street, Kermanshah, 67139546581 Iran; 2 School of Dentistry, Kermanshah University of Medical Sciences, Shariati Street, Kermanshah, 67139546581 Iran; 3 Department of Maxillofacial Radiology, School of Dentistry, Kermanshah University of Medical Sciences, Shariati Street, Kermanshah, 67139546581 Iran

**Keywords:** Cone-beam computed tomography, Iran, Bone screw, Orthodontic anchorage procedures

## Abstract

**Background:**

This study aimed to assess the interradicular distance and alveolar bone thickness of Persian adults with different sagittal skeletal patterns for miniscrew insertion using cone-beam computed tomography (CBCT).

**Methods:**

This cross-sectional study was conducted on maxillary and mandibular CBCT scans of 60 patients (18–35 years) in three groups (n = 20) of class I, II and III sagittal skeletal pattern. Anatomical and skeletal parameters were measured at 2, 4 and 6 mm apical to the cementoenamel junction (CEJ) by one examiner. The intra- and inter-class correlation coefficients were calculated to assess the intra, and interobserver reliability. Data were analyzed by ANOVA and Tukey’s test (alpha = 0.05).

**Results:**

The intra- and interobserver reliability were > 0.9 for all parameters. The largest inter-radicular distance in the maxilla was between the central incisors (1–1) in classes I and III, and between premolars (4–5) in class II patients. The largest inter-radicular distance in the mandible was between molar teeth (6–7) in all three classes. The buccal cortical plate thickness was maximum at the site of mandibular first and second molars (6–7). The posterior maxilla and mandible showed the maximum thickness of cancellous bone and alveolar process. Wide variations were noted in this respect between class I, II and III patients.

**Conclusions:**

The area with maximum inter-radicular distance and optimal alveolar bone thickness for miniscrew insertion varies in different individuals, depending on their sagittal skeletal pattern.

## Introduction

Orthodontic anchorage is defined as resistance against unwanted tooth movements [1]. Achieving maximum anchorage with no movement of the anchorage unit has always been a challenge in orthodontics, and success of treatment depends on the control and preparation of anchorage [2, 3]. Miniscrews, as the providers of skeletal anchorage, are gaining increasing popularity among orthodontists due to advantages such as provision of excellent anchorage, easy placement and retrieval, low cost, and small size [1, 4–6]. Nonetheless, miniscrews may become loose in the course of orthodontic treatment [5, 7]. Thus, their primary stability is a key factor in their success rate [8]. Several anatomical factors affect the stability of miniscrews and are important in their long-term success. These include skeletal factors such as cortical bone thickness, depth of insertion, and bone mineral density, soft tissue factors such as quality (mucosa versus the attached gingiva), tissue thickness, degree of tissue mobility, and frenal attachment, and presence of critical structures such as the roots, nerves, vasculature, sinus cavity, and nasal cavity [8, 9]. Of the abovementioned factors, more attention has been directed to skeletal factors because bone is mainly responsible for the miniscrew anchorage capacity.

Evidence shows that the anchorage capacity is mainly attributed to the quality and quantity of the cortical bone; however, cancellous bone probably plays a role in the stability of mini-screws as well [9, 10].

Bone thickness at the site of miniscrew insertion is another important factor to consider. Adequate bone is required for placement of miniscrews with the desired length to prevent perforation of the maxillary sinus or the nasal cavity [11]. Evidence shows that cortical bone thickness may be the most important factor in stability of miniscrews [7, 10]. The primary stability of miniscrews is significantly correlated with the trabecular bone thickness, indicating the significance of trabecular bone in miniscrew stability [12].

The overall available bone or bone depth is an important factor to consider in selection of a suitable site for placement of miniscrews. Adequate amount of bone is required for placement of miniscrews of a certain length in order to prevent contralateral perforation or invading the maxillary sinus or the nasal cavity, which would result in development of an oroantral communication [9].

The majority of the available literature regarding miniscrews have focused on their morphological parameters such as type, shape, diameter, and length, or assessed different anatomical sites for safe placement of miniscrews in inter-radicular spaces in the maxilla and mandible, reporting controversial results [2, 7, 11, 13–20]. A previous study discussed that vertical skeletal pattern can serve as an important factor in success of miniscrew placement in the posterior buccal areas [21]. The results of studies regarding the effect of facial height and different facial skeletal patterns on cortical plate thickness have been variable [3, 5, 22, 23]. A previous study compared the inter-radicular space and cortical bone thickness between two groups of Thai patients with class I and class III sagittal skeletal patterns. The results showed some differences between the two groups such that the alveolar process thickness, the buccal cortical plate, and the interradicular space in the maxilla were greater in class III patients while the mandibular alveolar process was wider in class I patients [24]. Al-Masri et al. [25] evaluated the thickness and density of bone in patients with different sagittal skeletal patterns and found greater alveolar bone thickness at the apical region of the buccal plate in class I and II patients compared with class III individuals. Also, the alveolar bone thickness at the cervical region of buccal cortical plate was greater in class I than class II patients.

Considering the controversial results regarding skeletal parameters related to safe and successful insertion of miniscrews, and limited number of studies comparing patients with different sagittal skeletal patterns in this respect [24, 25], this study aimed to assess the interradicular distance and alveolar bone thickness for miniscrew insertion in Persian adults with different sagittal skeletal patterns using cone-beam computed tomography (CBCT).

## Methods

This cross-sectional study evaluated CBCT scans (both jaws) of 60 patients between 18 and 35 years presenting to a private orthodontic office for orthodontic treatment. The CBCT scans had been taken for purposes not related to this study (such as preoperative assessment for septoplasty or third molar extraction). The study protocol was approved by the ethics committee of Kermanshah University of Medical Sciences (IR.KUMS.REC.1398.1017), and written informed consent was obtained from all patients for use of their CBCT scans in this study.

The minimum sample size was calculated to be 19 patients in each group according to a previous study by Al-Masri et al. [25] assuming the standard deviation of apical buccal thickness to be 1.42, d = 1.7, alpha = 0.05, and study power of 90%.

The inclusion criteria were absence of periodontal disease and alveolar bone loss, no history of previous orthodontic treatment, absence of severe skeletal discrepancy, no congenital missing (except for third molars), absence of severe crowding, and absence of developmental anomalies such as cleft lip and palate, or syndromes [2]. The cephalometric indices used for assessment of the sagittal pattern and the severity of skeletal discrepancy included the ANB angle and the Wits appraisal; according to which, the samples were divided into class I (ANB: 0°–4°; Wits 0 to − 1), class II (ANB > 4°, Wits > 0) and class III (ANB < 0°, Wits < − 1) groups.

The exclusion criterion was crowding > 5 mm [1].

The CBCT scans of all patients had been obtained in natural head position with their teeth in maximum intercuspation. The axial, sagittal and coronal sections were evaluated. The cross-sectional areas were evaluated on axial sections. The cementoenamel junction (CEJ) of the teeth was assessed on coronal sections, and the relationship of the jaws was assessed on lateral cephalograms retrieved from the orthodontic records of patients. All CBCT scans (15 × 15 cm) were obtained with 300 µm spatial resolution, 110 kV, and 78.59 mAs. The CBCT data in DICOM format were exported by NNT Viewer software to Mimics Medical Software version 21 (Materialise, Leuven, Belgium). To standardize the images and minimize errors in measurements, the images were reoriented in NNT Viewer such that the Frankfort horizontal plane and the line connecting the most inferior points in the inferior orbital rims were paralleled to the horizon. By doing so, the head position was standardized in all images, and all angles were measured relative to this line. The following hard tissue reference points were identified for cephalometric analysis:

- Point A: The deepest point on the curvature of the maxillary alveolar process between the anterior nasal spine and alveolar bone of the upper incisors
- Point B: The deepest point on the curvature of the mandibular alveolar process between the most superior point of the alveolar bone below the lower incisors and pogonion
- N: The anterior point of the intersection of nasal and frontal bones
- PO: The midpoint on the superior contour of the external auditory meatus
- Or: The most inferior point of the orbital rim

The ANB and Wits appraisal were used to assess the skeletal sagittal pattern of patients. Accordingly, the patients were assigned to three groups of class I, class II and class III by an experienced orthodontist. Class I patients had an ANB angle between 1° and 4°, with a Wits appraisal of − 1 to 0. Class II patients had an ANB angle > 4° with positive Wits appraisal, and class III patients had an ANB angle < 1° with negative Wits appraisal.

**Fig. 1:**
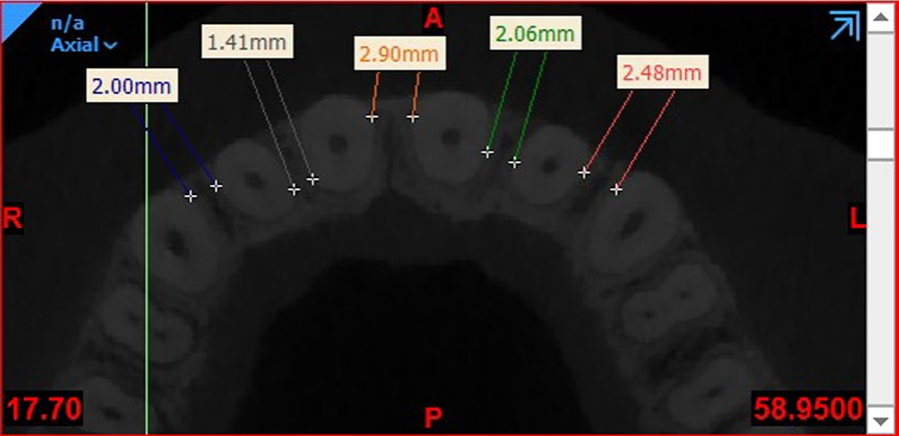
Measuring the inter-radicular distance
- Bone thickness: Axial sections at 2, 4 and 6 mm apical to the CEJ were used for measurement of bone thickness. On each section, the distance between the internal and external cortical plates was measured once at the thinnest part of the cortical bone and once at the widest part to measure the thickness of buccal and palatal/lingual cortical plates (Fig. 2) [27].

In this study, all measurements were made in both the maxilla and mandible at the site of central and lateral incisors and canine teeth for the anterior region, and first and second premolars and first and second molars in the posterior region at 2, 4 and 6 mm apical to the CEJ. The following anatomical parameters were measured in skeletal class I, II and III patients:

- Interradicular distance: Axial sections at 2, 4 and 6 mm apical to the CEJ were used for measurement of interradicular distance. On each axial section, the smallest distance between the adjacent roots was measured (Fig. 1) [26].

**Fig. 2:**
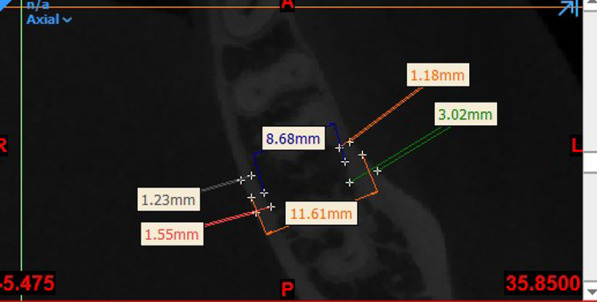
Measuring the thickness of cortical and cancellous bone and the alveolar process
- Cancellous bone thickness: The same images were used to measure the distance between the internal wall of the buccal cortical plate and the internal wall of the lingual/palatal cortical plate to determine the cancellous bone thickness (Fig. 2) [27, 28].
- Alveolar process thickness: It was measured as the distance between the outermost point on the buccal to the outermost point on the palatal/lingual surface at the center of the distance between two adjacent teeth (Fig. 2) [27].

Distance between the maxillary sinus floor and CEJ of the teeth: Cross-sectional images of inter-radicular areas, from canine to second molar of each quadrant of the maxilla, were used to measure the shortest distance between the maxillary sinus floor and CEJ of the teeth by drawing a panoramic curve on axial images [26].

The measurements were made by one examiner. To assess the intraobserver reliability, 20 CBCT scans were randomly selected and measurements were repeated on them after 2 weeks. The measured values were compared with the primary values and the intraclass correlation coefficient was calculated.

Data were analyzed using SPSS version 16. Since all variables had a normal distribution (*P* > 0.05), one-way ANOVA was used to compare each value among class I, class II and class III patients. In case of presence of a significant difference among the three groups in a variable, pairwise comparisons were performed by the Tukey’s post hoc test. Level of significance was set at 0.05.

## Results

A total of 60 CBCT scans were evaluated. There were 13 females and 7 males in each skeletal class. The mean age of patients was 29.15 ± 6.18, 24.25 ± 6.62, and 26.70 ± 6.04 years in class I, class II, and class III patients, respectively. The difference in the mean age was not significant among the three groups (*P* = 0.056). The inter- and intraclass correlation coefficients were > 0.90 for all parameters, indicating excellent inter- and intraobserver reliability.

### Interradicular distance

The maximum interradicular distance at different levels from the CEJ in the maxilla was as follows:

- At 2 mm level: The maximum interradicular distance was noted at the site of 1–1 in class I and III patients and at the site of 5–6 in class II patients.
- At 4 mm level: The maximum interradicular distance was noted at the site of 1–1 in class I and III patients and at the site of 4–5 in class II patients.
- At 6 mm level: The maximum interradicular distance was noted at the site of 1–1 in class I and III patients and at the site of 4–5 in class II patients.
- Entire maxilla: The maximum interradicular distance was noted at the site of 1–1 at 6 mm from the CEJ in class I patients (3.85 mm).

The maximum interradicular distance at different levels from the CEJ in the mandible was as follows:

- At 2 mm level: The maximum interradicular distance was noted at the site of 6–7 in class I and II patients and at the site of 5–6 in class III patients.
- At 4 mm level: The maximum interradicular distance was noted at the site of 6–7 in all three classes.
- At 6 mm level: The maximum interradicular distance was noted at the site of 6–7 in all three classes.
- Entire mandible: The maximum interradicular distance was noted at the site of 6–7 at 6 mm distance from the CEJ in class II patients (5.02 mm).

One-way ANOVA showed significant differences in the mean interradicular distance among the three skeletal classes (*P* < 0.05). Tables 1 and 2 shows descriptive statistics and compares the mean interradicular distance in the maxilla and mandible at different levels from the CEJ in class I, II, and III patients.

**Table 1:** Descriptive statistics (mean and standard deviation) and comparison of the mean interradicular distance in the maxilla at different levels from the CEJ in class I, II, and III patients

HightLocation	Level										*P* value tukey test								
		2 mm			4 mm			6 mm			2 mm			4 mm			6 mm		
		I	II	III	I	II	III	I	II	III	I–II	II–III	I–III	I–II	II–III	I–III	I–II	II–III	I–III
*Maxilla*																			
1–1	Mean	2.39	2.54	2.13	3.15	2.95	2.82	3.85	3.32	3.50	0.86	0.33	0.64	0.82	0.91	0.58	0.35	0.88	0.63
	SD	0.80	0.62	1.17	0.75	0.87	1.44	0.96	1.12	1.48									
1–2	Mean	1.43	1.47	1.32	1.63	1.77	1.77	1.98	2.21	1.98	0.95	0.48	0.67	0.71	0.99	0.71	0.52	0.52	0.99
	SD	0.25	0.57	0.42	0.36	0.62	0.62	0.45	0.70	0.81									
2–3	Mean	1.81	2.31	1.61	2.34	2.66	2.12	2.82	2.94	2.38	0.04	0.03	0.58	0.25	0.02	0.52	0.85	0.03	0.12
	SD	0.53	0.76	0.58	0.45	0.68	0.72	0.50	0.81	0.77									
3–4	Mean	1.72	1.51	1.26	1.93	1.81	1.59	2.15	2.19	1.94	0.37	0.25	0.01	0.85	0.57	0.28	0.99	0.71	0.79
	SD	0.46	0.50	0.50	0.53	0.76	0.74	0.70	1.25	1.01									
4–5	Mean	1.96	2.46	1.93	2.53	2.99	2.39	2.87	3.42	2.46	0.04	0.02	0.98	0.04	0.02	0.73	0.10	0.02	0.28
	SD	0.67	0.67	0.52	0.65	0.58	0.56	0.72	1.01	0.74									
5–6	Mean	2.03	2.62	1.99	2.33	2.95	2.48	2.67	3.25	2.80	0.02	0.02	0.96	0.01	0.08	0.79	0.05	0.15	0.86
	SD	0.42	0.60	0.63	0.37	0.86	0.74	0.67	0.78	0.84									
6–7	Mean	1.74	1.84	1.44	1.74	1.90	1.55	1.72	1.58	1.52	0.82	0.04	0.16	0.70	0.22	0.66	0.80	0.95	0.63
	SD	0.53	0.55	0.48	0.62	0.75	0.6	0.74	0.60	0.73

**Table 2:** Descriptive statistics (mean and standard deviation) and comparison of the mean interradicular distance in the mandible at different levels from the CEJ in class I, II, and III patients

Hight Location		Level									*P* value tukey test								
		2 mm			4 mm			6 mm			2 mm			4 mm			6 mm		
		I	II	III	I	II	III	I	II	III	I–II	II–III	I–III	I–II	II–III	I–III	I–II	II–III	I–III
*Mandible*																			
1–1	Mean	1.49	1.50	1.59	1.75	1.63	1.70	2.05	1.78	1.75	0.99	0.88	0.83	0.82	0.94	0.96	0.51	0.99	0.45
	SD	0.49	0.63	0.58	0.51	0.80	0.65	0.59	1.01	0.66									
1–2	Mean	1.15	1.47	1.51	1.34	1.47	1.72	1.44	1.60	1.95	0.02	0.94	0.01	0.64	0.22	0.03	0.95	0.06	0.03
	SD	0.31	0.47	0.37	0.37	0.54	0.44	0.48	0.73	0.60									
2–3	Mean	1.05	1.55	1.59	1.40	1.84	1.87	1.77	2.19	2.47	0.01	0.94	0.01	0.01	0.97	0.01	0.05	0.26	0.01
	SD	0.39	0.26	0.47	0.42	0.36	0.60	0.44	0.45	0.74									
3–4	Mean	1.64	1.87	1.85	1.99	2.23	2.12	2.27	2.54	2.49	0.26	0.98	0.33	0.36	0.81	0.73	0.38	0.96	0.52
	SD	0.43	0.47	0.47	0.53	0.50	0.63	0.47	0.58	0.71									
4–5	Mean	2.13	2.52	2.03	2.75	3.32	2.54	3.32	3.99	3.28	0.01	0.01	0.74	0.01	0.01	0.36	0.01	0.01	0.96
	SD	0.52	0.42	0.24	0.56	0.52	0.34	0.62	0.51	0.46									
5–6	Mean	2.52	2.87	2.56	2.92	3.68	3.05	3.25	4.18	3.56	0.03	0.07	0.95	0.01	0.02	0.79	0.03	0.06	0.48
	SD	0.56	0.45	0.22	0.58	0.85	0.39	0.69	1.23	0.46									
6–7	Mean	3.23	3.32	2.50	3.80	4.17	3.38	4.27	5.02	3.87	0.93	0.01	0.01	0.55	0.07	0.47	0.25	0.04	0.67
	SD	1.13	0.74	0.28	1.60	0.84	0.72	1.96	1.19	1.15									

### Alveolar process thickness

The maximum alveolar process thickness in the maxilla in class I, II and III patients was at the site of 6–7 at 2, 4 and 6 mm levels. Also, the maximum thickness of alveolar process in the entire maxilla was at the site of 6–7 at 6 mm level in class II patients (14.58 mm).

The maximum alveolar process thickness in the mandible in class I, II and III patients was at the site of 6–7 at 2, 4 and 6 mm levels. Also, the maximum thickness of alveolar process in the entire mandible was at the site of 6–7 at 6 mm level in class II patients (12.17 mm).

The maximum alveolar process thickness was significantly different among the three groups in both the maxilla (*P* < 0.05) and mandible (*P* < 0.05). Tables 3 and 4 shows descriptive statistics and compares the mean alveolar process thickness in the maxilla and mandible at different levels from the CEJ in class I, II and III patients.

**Table 3:** Descriptive statistics (mean and standard deviation) and comparison of the mean alveolar process thickness in the maxilla at different levels from the CEJ in class I, II and III patients

Hight Location		Level									*P* value tukey test								
		2 mm			4 mm			6 mm			2 mm			4 mm			6 mm		
		I	II	III	I	II	III	I	II	III	I–II	II–III	I–III	I–II	II–III	I–III	I–II	II–III	I–III
*Maxilla*																			
1–1	Mean	6.14	5.06	4.85	6.78	6.23	6.51	8.65	6.60	7.26	0.07	0.90	0.02	0.45	0.81	0.82	0.01	0.62	0.13
	SD	1.32	1.11	2.06	1.10	1.24	1.87	2.28	1.86	2.55									
1–2	Mean	6.72	5.97	6.34	8.35	7.42	7.67	9.18	8.04	7.86	0.26	0.71	0.70	0.04	0.77	0.18	0.02	0.89	0.01
	SD	1.37	1.13	1.87	1.08	0.46	1.68	1.26	0.85	1.76									
2–3	Mean	6.59	5.44	5.88	7.99	7.30	7.83	9.11	7.94	8.42	0.02	0.57	0.23	0.26	0.44	0.92	0.01	0.48	0.21
	SD	1.30	1.08	1.65	1.39	1.27	1.45	1.39	1.14	1.34									
3–4	Mean	7.39	7.08	6.81	8.92	8.75	8.38	9.14	9.17	8.68	0.58	0.66	0.16	0.85	0.51	0.23	0.99	0.36	0.41
	SD	0.96	0.88	1.11	1.08	0.78	1.22	1.2	0.92	1.20									
4–5	Mean	8.58	8.66	8.00	9.30	9.47	8.68	9.27	9.41	8.27	0.98	0.31	0.41	0.86	0.05	0.15	0.90	0.01	0.01
	SD	1.76	1.04	1.36	1.15	0.72	1.17	0.93	0.63	1.39									
5–6	Mean	10.48	9.58	8.14	10.54	11.25	9.81	10.69	11.38	9.64	0.15	0.01	0.01	0.21	0.03	0.19	0.19	0.01	0.02
	SD	1.43	1.39	1.69	1.35	1.29	1.31	0.97	0.99	1.67									
6–7	Mean	11.71	12.4	9.65	12.8	13.86	11.37	13.37	14.58	12.3	0.41	0.01	0.03	0.12	0.01	0.02	0.03	0.01	0.07
	SD	2.11	2.13	1.19	1.80	1.54	1.71	1.25	1.52	1.67

**Table 4:** Descriptive statistics (mean and standard deviation) and comparison of the mean alveolar process thickness in the mandible at different levels from the CEJ in class I, II and III patients

Hight Location		Level									*P* value tukey test								
		2 mm			4 mm			6 mm			2 mm			4 mm			6 mm		
		I	II	III	I	II	III	I	II	III	I–II	II–III	I–III	I–II	II–III	I–III	I–II	II–III	I–III
*Mandible*																			
1–1	Mean	5.15	4.28	3.95	5.50	5.87	4.84	5.75	6.21	5.00	0.06	0.67	0.01	0.52	0.01	0.13	0.37	0.03	0.08
	SD	1.27	1.28	1.05	1.18	1.14	0.82	1.06	1.18	1.02									
1–2	Mean	5.94	5.26	4.80	6.78	7.02	5.77	6.49	6.84	5.74	0.16	0.44	0.01	0.72	0.01	0.02	0.53	0.03	0.06
	SD	1.32	1.29	0.86	1.15	1.12	0.61	1.09	0.91	1.06									
2–3	Mean	6.73	6.36	5.26	7.53	7.52	6.76	7.03	6.80	6.40	0.60	0.01	0.01	0.95	0.10	0.09	0.83	0.58	0.26
	SD	1.49	0.92	1.17	1.63	0.76	0.87	1.26	1.30	1.23									
3–4	Mean	6.82	5.16	5.13	7.89	7.46	6.91	8.30	8.11	6.92	0.01	0.99	0.04	0.52	0.36	0.04	0.81	0.01	0.01
	SD	1.08	1.21	0.69	1.52	1.38	0.74	1.28	0.58	0.99									
4–5	Mean	6.80	6.46	5.46	7.66	7.78	6.68	8.32	7.83	7.00	0.59	0.01	0.01	0.89	0.01	0.02	0.30	0.04	0.01
	SD	0.73	1.37	1.13	1.00	0.67	0.75	1.37	0.66	0.99									
5–6	Mean	8.20	7.77	6.62	8.90	8.89	7.80	9.51	9.09	7.96	0.42	0.01	0.01	0.95	0.03	0.03	0.49	0.02	0.02
	SD	0.79	1.54	0.73	1.06	0.92	0.98	1.23	1.23	0.97									
6–7	Mean	9.27	9.63	8.06	10.33	11.15	9.49	11.87	12.17	10.7	0.70	0.01	0.02	0.21	0.02	0.20	0.80	0.01	0.06
	SD	1.58	1.06	1.60	1.71	0.95	1.77	1.75	1.14	1.66									

### Cancellous bone thickness

The maximum mean cancellous bone thickness in the maxilla was at the site of 6–7 at 2, 4 and 6 mm levels in all three skeletal classes. The maximum mean cancellous bone thickness in the entire maxilla was at the site of 6–7 at 6 mm level in class II patients (12.60 mm).

The maximum mean cancellous bone thickness in the mandible was at the site of 6–7 at 2, 4 and 6 mm levels in all three skeletal classes. The maximum mean cancellous bone thickness in the entire mandible was at the site of 6–7 at 6 mm level in class II patients (9.03 mm).

Significant differences were noted in the mean cancellous bone thickness in both the maxilla and mandible among the three classes (*P* < 0.05). Tables 5 and 6 shows descriptive statistics and compares the mean cancellous bone thickness in the maxilla and mandible in class I, II and III patients.

**Table 5:** Descriptive statistics (mean and standard deviation) and comparison of the mean cancellous bone thickness in the maxilla in class I, II and III patients

Hight Location		Level									*P* value tukey test								
		2 mm			4 mm			6 mm			2 mm			4 mm			6 mm		
		I	II	III	I	II	III	I	II	III	I–II	II–III	I–III	I–II	II–III	I–III	I–II	II–III	I–III
*Maxilla*																			
1–1	Mean	3.80	2.76	2.92	4.52	4.13	4.51	5.81	4.59	5.20	0.06	0.92	0.13	0.59	0.61	0.99	0.10	0.55	0.56
	SD	1.33	1.02	1.78	0.79	1.04	1.80	1.73	1.49	2.29									
1–2	Mean	4.16	3.89	4.21	5.85	5.51	5.36	6.50	5.79	5.65	0.81	0.75	0.99	0.48	0.87	0.23	0.15	0.92	0.07
	SD	1.24	1.23	1.61	0.78	0.47	1.35	0.92	0.88	1.62									
2–3	Mean	4.30	3.47	3.86	5.43	5.50	5.65	6.40	5.87	6.14	0.14	0.64	0.57	0.98	0.92	0.84	0.42	0.79	0.81
	SD	1.44	1.04	1.59	1.00	1.45	1.27	1.18	1.30	1.52									
3–4	Mean	5.12	5.00	4.87	6.29	6.57	6.25	6.58	7.15	6.32	0.93	0.93	0.76	0.67	0.59	0.99	0.22	0.04	0.72
	SD	0.97	1.14	1.23	1.01	1.00	1.05	0.96	1.17	1.06									
4–5	Mean	6.31	6.73	6.28	7.11	7.62	6.49	6.92	7.55	6.12	0.61	0.56	0.99	0.30	0.04	0.16	0.10	0.01	0.03
	SD	1.69	1.13	1.31	1.12	0.84	1.22	0.91	0.85	1.13									
5–6	Mean	7.87	7.34	6.25	8.40	9.53	7.64	8.46	9.81	7.63	0.56	0.09	0.03	0.02	0.01	0.16	0.02	0.03	0.07
	SD	1.63	1.42	1.78	1.36	1.40	1.11	0.95	1.10	1.39									
6–7	Mean	9.12	10.49	7.49	10.7	12.14	9.54	11.33	12.6	10.3	0.07	0.05	0.02	0.02	0.01	0.07	0.02	0.01	0.11
	SD	2.23	2.29	1.01	1.85	1.53	1.49	1.31	1.57	1.53

**Table 6:** Descriptive statistics (mean and standard deviation) and comparison of the mean cancellous bone thickness in the mandible in class I, II and III patients

Hight Location		Level									*P* value tukey test								
		2 mm			4 mm			6 mm			2 mm			4 mm			6 mm		
		I	II	III	I	II	III	I	II	III	I–II	II–III	I–III	I–II	II–III	I–III	I–II	II–III	I–III
*Mandible*																			
1–1	Mean	2.83	2.72	2.30	2.88	4.06	2.82	3.24	4.30	2.88	0.93	0.42	0.25	0.01	0.02	0.97	0.02	0.04	0.43
	SD	1.10	1.19	0.79	0.90	1.16	0.50	0.89	1.10	0.73									
1–2	Mean	3.46	3.21	3.07	4.56	5.19	3.80	3.96	4.90	3.69	0.76	0.91	0.51	0.08	0.04	0.02	0.01	0.03	0.66
	SD	1.15	1.39	0.75	0.96	1.02	0.71	0.88	0.99	1.07									
2–3	Mean	4.13	4.30	3.06	4.76	5.26	4.10	4.24	4.89	3.83	0.87	0.02	0.03	0.22	0.01	0.08	0.15	0.03	0.43
	SD	1.32	0.82	1.10	1.16	0.80	0.84	0.86	1.16	1.16									
3–4	Mean	4.18	3.21	2.95	5.02	4.99	4.29	4.99	5.76	4.19	0.01	0.72	0.02	0.99	0.09	0.07	0.03	0.01	0.02
	SD	1.00	1.33	0.84	1.04	1.27	0.68	1.03	0.88	0.85									
4–5	Mean	4.56	4.53	3.37	4.88	5.44	4.15	5.11	5.40	4.08	0.99	0.04	0.03	0.13	0.01	0.03	0.61	0.01	0.04
	SD	0.90	1.21	1.15	0.99	0.83	0.86	1.01	0.94	0.96									
5–6	Mean	5.55	6.12	4.22	6.27	6.91	5.39	6.31	6.56	5.18	0.28	0.01	0.02	0.05	0.01	0.01	0.02	0.01	0.03
	SD	1.15	1.39	0.94	0.79	0.78	0.94	0.79	1.37	0.67									
6–7	Mean	6.61	7.61	5.78	7.25	8.23	6.57	7.78	9.03	6.95	0.05	0.01	0.10	0.05	0.01	0.23	0.04	0.01	0.07
	SD	1.16	1.16	1.43	1.23	0.91	1.67	1.15	0.83	1.43									

### Buccal cortical plate thickness

The maximum mean buccal cortical plate thickness in the maxilla was at the site of 5–6 in classes I and II, and 6–7 in class III at 2 mm level, 5–6 in classes II and III and 1–1 in class I at 4 mm level, and 1–1 in class I, 1–2 in class II and 5–6 in class III at 6 mm level from the CEJ. In the entire maxilla, the maximum mean buccal cortical plate thickness was recorded at the site of 1–1 at 6 mm level in class I patients (1.13 mm).

The maximum mean cortical plate thickness in the mandible was at the site of 6–7 in all three classes and at all levels from the CEJ. In the entire mandible, the maximum mean thickness was noted at the site of 6–7 at 6 mm level in class I patients (2.13 mm).

Since the difference was significant in the mean buccal cortical plate thickness among the three classes in both the maxilla (*P* < 0.05) and mandible (*P* < 0.05), Tables 7 and 8 shows descriptive statistics and compares the mean buccal cortical plate thickness in the maxilla and mandible at different levels from the CEJ in the three skeletal classes.

**Table 7:** Descriptive statistics (mean and standard deviation) and comparison of the mean buccal cortical plate thickness in the maxilla at different levels from the CEJ in the three skeletal classes

Hight Location		Level									*P* value tukey test								
		2 mm			4 mm			6 mm			2 mm			4 mm			6 mm		
		I	II	III	I	II	III	I	II	III	I–II	II–III	I–III	I–II	II–III	I–III	I–II	II–III	I–III
*Maxilla*																			
1–1	Mean	1.03	0.97	0.79	1.11	0.77	0.79	1.13	0.68	0.79	0.81	0.20	0.05	0.02	0.98	0.03	0.19	0.53	0.04
	SD	0.30	0.38	0.26	0.40	0.14	0.17	0.54	0.12	0.19									
1–2	Mean	0.91	0.83	0.80	0.97	0.75	0.85	1.07	0.88	0.87	0.53	0.87	0.27	0.02	0.39	0.23	0.03	0.98	0.02
	SD	0.22	0.26	0.19	0.26	0.14	0.25	0.30	0.24	0.12									
2–3	Mean	0.92	0.80	0.79	0.93	0.78	0.79	0.95	0.79	0.88	0.09	0.99	0.07	0.06	0.99	0.08	0.13	0.54	0.65
	SD	0.16	0.14	0.22	0.26	0.17	0.17	0.22	0.18	0.32									
3–4	Mean	1.13	0.71	0.81	1.04	0.74	0.94	0.95	0.77	0.79	0.01	0.30	0.02	0.03	0.04	0.26	0.01	0.93	0.03
	SD	0.23	0.17	0.24	0.18	0.10	0.26	0.26	0.11	0.17									
4–5	Mean	1.02	0.81	0.82	1.01	0.77	0.85	0.88	0.77	0.82	0.02	0.98	0.03	0.03	0.47	0.03	0.10	0.58	0.51
	SD	0.15	0.20	0.16	0.27	0.10	0.20	0.14	0.17	0.16									
5–6	Mean	1.08	1.01	0.83	0.94	0.81	1.01	0.92	0.74	0.89	0.71	0.15	0.02	0.20	0.03	0.66	0.01	0.04	0.85
	SD	0.27	0.33	0.25	0.24	0.13	0.30	0.19	0.11	0.24									
6–7	Mean	1.08	0.96	0.99	0.99	0.78	0.83	1.02	0.85	0.73	0.44	0.97	0.57	0.03	0.74	0.01	0.01	0.16	0.04
	SD	0.29	0.36	0.23	0.23	0.14	0.17	0.26	0.16	0.12

**Table 8:** Descriptive statistics (mean and standard deviation) and comparison of the mean buccal cortical plate thickness in the mandible at different levels from the CEJ in the three skeletal classes

Hight Location		Level									*P* value tukey test								
		2 mm			4 mm			6 mm			2 mm			4 mm			6 mm		
		I	II	III	I	II	III	I	II	III	I–II	II–III	I–III	I–II	II–III	I–III	I–II	II–III	I–III
*Mandible*																			
1–1	Mean	0.86	0.72	0.68	0.93	0.83	0.81	0.85	0.80	0.82	0.07	0.73	0.01	0.58	0.97	0.45	0.74	0.92	0.93
	SD	0.28	0.16	0.10	0.46	0.20	0.17	0.26	0.18	0.20									
1–2	Mean	0.89	0.86	0.74	0.96	0.78	0.83	0.94	0.77	0.74	0.85	0.14	0.04	0.03	0.73	0.17	0.01	0.91	0.03
	SD	0.23	0.18	0.10	0.29	0.15	0.20	0.18	0.17	0.19									
2–3	Mean	0.96	0.87	0.81	0.99	0.80	0.84	1.03	0.74	0.84	0.50	0.75	0.16	0.02	0.84	0.09	0.03	0.33	0.01
	SD	0.36	0.16	0.20	0.26	0.19	0.19	0.28	0.11	0.20									
3–4	Mean	0.97	0.83	0.90	0.95	0.85	0.91	1.10	0.76	0.94	0.29	0.73	0.73	0.14	0.50	0.70	0.01	0.02	0.07
	SD	0.30	0.28	0.32	0.19	0.13	0.17	0.29	0.12	0.20									
4–5	Mean	0.89	0.84	0.93	1.07	0.89	0.98	1.27	0.89	1.00	0.75	0.45	0.87	0.03	0.38	0.40	0.04	0.17	0.02
	SD	0.23	0.14	0.26	0.24	0.15	0.23	0.24	0.12	0.18									
5–6	Mean	1.15	0.73	1.02	1.14	0.88	1.09	1.36	0.90	1.21	0.01	0.03	0.28	0.03	0.04	0.76	0.02	0.03	0.30
	SD	0.32	0.16	0.24	0.30	0.17	0.27	0.34	0.16	0.36									
6–7	Mean	1.44	1.12	1.20	1.78	1.34	1.48	2.13	1.59	1.92	0.02	0.75	0.09	0.01	0.63	0.11	0.02	0.6	0.27
	SD	0.37	0.35	0.33	0.60	0.34	0.41	0.55	0.32	0.41									

### Palatal cortical plate thickness in the maxilla

The maximum mean palatal cortical plate thickness in the maxilla was noted at the site of 1–1 in classes II and III and 1–2 in class I patients at 2 mm level, at the site of 2–3 in classes I and III and at the site of 3–4 in class II at 4 mm level, and at the site of 3–4 in class I, 1–2 in class II, and 2–3 in class III at 6 mm level from the CEJ. In the entire maxilla, the maximum palatal cortical plate thickness was noted at the site of 3–4 at 6 mm level in class I patient (1.63 mm). Table 9 compares the mean palatal cortical plate thickness in the maxilla at different levels from the CEJ in the three skeletal classes.

**Table 9:** Descriptive statistics (mean and standard deviation) and comparison of the mean palatal cortical plate thickness in the maxilla at different levels from the CEJ in the three skeletal classes

Hight Location		Level									*P* value tukey test								
		2 mm			4 mm			6 mm			2 mm			4 mm			6 mm		
		I	II	III	I	II	III	I	II	III	I–II	II–III	I–III	I–II	II–III	I–III	I–II	II–III	I–III
*Maxilla*																			
1–1	Mean	1.24	0.88	1.10	1.29	0.87	0.99	1.27	0.93	0.98	0.03	0.10	0.39	0.03	0.23	0.16	0.04	0.86	0.03
	SD	0.41	0.30	0.24	0.29	0.17	0.21	0.40	0.39	0.21									
1–2	Mean	1.39	0.87	1.08	1.49	0.89	1.17	1.54	1.15	1.15	0.03	0.14	0.01	0.01	0.04	0.01	0.02	0.99	0.04
	SD	0.54	0.14	0.19	0.54	0.13	0.24	0.56	0.31	0.17									
2–3	Mean	1.34	0.86	1.01	1.50	0.97	1.16	1.61	1.04	1.24	0.01	0.39	0.01	0.01	0.11	0.02	0.02	0.10	0.01
	SD	0.48	0.20	0.34	0.45	0.18	0.18	0.48	0.16	0.13									
3–4	Mean	1.14	0.85	0.83	1.45	1.00	1.15	1.63	1.14	1.30	0.02	0.97	0.01	0.01	0.46	0.04	0.02	0.28	0.03
	SD	0.39	0.26	0.32	0.47	0.27	0.36	0.30	0.40	0.29									
4–5	Mean	1.14	0.73	0.76	1.20	0.90	1.05	1.27	0.96	1.21	0.02	0.85	0.03	0.02	0.07	0.06	0.03	0.02	0.75
	SD	0.24	0.14	0.19	0.26	0.13	0.21	0.37	0.23	0.22									
5–6	Mean	1.29	0.95	0.92	1.17	0.98	0.96	1.15	0.90	1.13	0.04	0.93	0.01	0.03	0.95	0.01	0.02	0.01	0.89
	SD	0.45	0.18	0.23	0.21	0.30	0.18	0.24	0.15	0.14									
6–7	Mean	1.38	0.81	0.94	1.12	0.83	0.89	1.03	0.98	0.98	0.01	0.68	0.02	0.01	0.72	0.01	0.75	0.99	0.76
	SD	0.77	0.19	0.18	0.32	0.21	0.16	0.21	0.21	0.22									

### Lingual cortical plate thickness in the mandible

The maximum mean lingual cortical plate thickness in the mandible was noted at the site of 2–3 in all three classes at 2 mm level, at the site of 3–4 in all three classes at 4 mm level, and at the site of 3–4 in class I, 6–7 in class II, and 3–4 in class III patients at 6 mm level from the CEJ. In the entire mandible, the maximum thickness was noted at the site of 3–4 at 6 mm level in class I patients (2.07). Table 10 compares the lingual cortical plate thickness in the mandible at different levels from the CEJ in the three skeletal classes.

**Table 10:** Descriptive statistics (mean and standard deviation) and comparison of lingual cortical plate thickness in the mandible at different levels from the CEJ in the three skeletal classes

Hight Location		Level									*P* value tukey test								
		2 mm			4 mm			6 mm			2 mm			4 mm			6 mm		
		I	II	III	I	II	III	I	II	III	I–II	II–III	I–III	I–II	II–III	I–III	I–II	II–III	I–III
*Mandible*																			
1–1	Mean	1.21	0.73	0.77	1.53	0.87	1.10	1.63	0.88	1.20	0.03	0.88	0.04	0.02	0.15	0.02	0.02	0.01	0.01
	SD	0.37	0.15	0.20	0.54	0.25	0.27	0.35	0.32	0.35									
1–2	Mean	1.27	0.79	0.88	1.22	0.81	1.01	1.48	0.87	1.18	0.02	0.57	0.01	0.03	0.03	0.03	0.04	0.03	0.02
	SD	0.44	0.15	0.14	0.34	0.16	0.21	0.48	0.20	0.28									
2–3	Mean	1.54	0.92	1.03	1.62	1.16	1.43	1.93	1.09	1.65	0.03	0.77	0.04	0.01	0.05	0.22	0.01	0.01	0.17
	SD	0.61	0.25	0.20	0.50	0.20	0.31	0.61	0.14	0.53									
3–4	Mean	1.53	0.81	1.02	1.83	1.23	1.54	2.07	1.17	1.67	0.04	0.13	0.01	0.02	0.10	0.12	0.02	0.01	0.02
	SD	0.47	0.18	0.29	0.62	0.38	0.34	0.41	0.26	0.27									
4–5	Mean	1.35	0.90	1.00	1.71	1.21	1.36	2.06	1.36	1.66	0.02	0.57	0.02	0.01	0.41	0.01	0.03	0.03	0.04
	SD	0.39	0.20	0.27	0.42	0.30	0.38	0.42	0.17	0.19									
5–6	Mean	1.34	0.75	1.02	1.52	1.01	1.13	1.84	1.24	1.42	0.01	0.02	0.03	0.01	0.57	0.04	0.02	0.01	0.02
	SD	0.35	0.19	0.34	0.48	0.23	0.34	0.45	0.23	0.30									
6–7	Mean	1.12	0.86	1.00	1.49	1.13	1.37	1.81	1.41	1.50	0.02	0.30	0.40	0.01	0.03	0.42	0.02	0.03	0.04
	SD	0.27	0.19	0.37	0.33	0.25	0.30	0.41	0.24	0.31									

### Distance between the maxillary sinus floor and CEJ of posterior teeth

Table 11 compares the mean distance between the maxillary sinus floor and CEJ of posterior teeth among the three classes. The maximum mean distance in the posterior region at the left side was noted at the sites of 3–4, 4–5, and 5–6 in class I, and 3–4 in class II and III patients.

**Table 11:** Descriptive statistics (mean and standard deviation) and comparison of the mean distance between the maxillary sinus floor and CEJ of posterior teeth among the three classes

Sagittal skeletal patterns		Left				*P* value	Right				*P* value
		L 6–7	L 5–6	L 4–5	L 3–4		R 3–4	R 4–5	R 5–6	R 6–7	
Class I	Mean	12.63	15.60*	21.25*	22.73*	.003	23.89*	21.56*	16.58	13.12	.000
	SD	2.95	4.16	5.47	9.74		8.02	5.56	4.28	2.88	
Class II	Mean	12.72	11.87	16.70	21.88*	.000	20.72*	13.97	11.33	11.42	.000
	SD	3.41	5.04	6.55	6.84		7.25	6.57	4.81	3.57	
Class III	Mean	12.27	16.35	20.67	27.03*	.000	27.11*	20.45	15.10	11.91	.000
	SD	2.98	6.50	6.32	6.69		6.64	6.61	5.61	3.00	

Repeated measures ANOVA was used. Means with * are significantly greater than other means at the same distance

SD, standard deviation

The maximum mean distance in the posterior region at the right side was noted at the sites of 3–4, and 4–5 in class I, 3–4 in class II, and 3–4 in class III patients. In general, the maximum distance was noted in the posterior maxilla and at the site of 3–4 in all three classes. The minimum distance was found in the posterior maxilla at the site of 6–7 in classes I and III and at the site of 5–6 in class II patients.

## Discussion

Safe insertion and no traumatization of the adjacent anatomical structures such as the roots, blood vessels, nerves, nasal cavity, and the maxillary sinus are among the most important factors to consider in miniscrew insertion [11]. Also, the quality and quantity of the bone play a fundamental role in success of miniscrew placement [29]. This study assessed the hard tissue anatomical parameters to find the best site for miniscrew placement in different sagittal skeletal classes. The results indicated that irrespective of the most important bone properties related to miniscrew stability, the safest area for miniscrew placement in the mandible of class I, class II and class III patients is the area between the first and second molar teeth. However, determining the safest area for miniscrew placement in the maxilla is difficult.

Previous studies on this topic used different anatomical landmarks such as the CEJ [24] or alveolar crest [14, 30] as the reference points. CEJ was used for this purpose in the present study due to its constant position, visibility, and easy access by the examiner.

With respect to the importance of inter-radicular distance in miniscrew placement, Mohammed et al. [19] reported the lower clinical success rate of interradicular mini-screws due to their contact with the roots. A minimum of 1 mm clearance from the alveolar bone around the miniscrew has been suggested for periodontal health [7]. Thus, the interradicular distance should be > 3 mm for miniscrew placement [30, 31].

In the present study, the maximum interradicular distance in the maxilla at all axial levels was recorded at the site of 1–1 in classes I and II and 4–5 in class II at 6 mm level from the CEJ. Also, in the entire maxilla, the maximum interradicular distance was noted at the site of 1–1 at 6 mm level in class I patients. The maximum interradicular distance in the maxilla was noted at the site of 5–6 in studies by Chaimanee et al. [31] in all three skeletal patterns, Khumsarn et al. [24] in classes I and II, and Park and Cho [32]. Moreover, Poggio et al. [30] reported maximum interradicular distance in the maxilla at the site of 5–6 in the palatal and 4–5, and 3–4 in the buccal surface. Their findings were partly in line with the findings of the present study in class II patients. Fayed et al. [27] reported the maximum interradicular distance to be at the site of 1–1 in the buccal in the anterior maxilla and at the site of 5–6 in both buccal and palatal sides in the posterior maxilla, which was in agreement with the present findings in classes I and III. Lim et al. [14] reported the largest interradicular distance at the sites of 4–5, and 5–6 in the buccal side.

Our results revealed that in the mandible, the maximum interradicular distance was at the site of 6–7 in all three classes, and the maximum value was recorded in class II individuals. This finding was in agreement with the results of Hu et al. [33]. Our results were similar to the findings of Khumsarn et al. [24] and Chaimanee et al. [31] at the sites of 4–5 and 6–7, Fayed et al. [27] and Lim et al. [14] at the sites of 4–5 and 5–6, and in contrast to those of Poggio et al. [30] at the site of 4–5. Such variations in the results can be attributed to different methodologies. For example, many studies [13, 30, 31] used the alveolar crest as the reference point, which is not reliable and can be affected by periodontal disease. Also, some others did not use 3D CBCT scans for such measurements [31, 33] or used different sections such as sagittal sections [30, 32]. Moreover, different methods of measurement may explain some differences in the results [2, 24, 27, 30, 33]. On the other hand, some previous studies [2, 14, 27, 30, 32, 33] did not classify the patients based on their sagittal skeletal pattern. Different races and ethnicities can also be responsible for the variations in the results. Furthermore, the distance between the axial level and the reference point (used for assessment of skeletal structures) can greatly affect the results [30–32]. For example, the cortical thickness [34] and the interradicular distance [35] are both greater in more apical levels.

According to Motoyoshi [36] presence of an area with cortical bone thickness of at least 1 mm is imperative to increase the success rate of mini-implant placement. The present results revealed the maximum buccal cortical plate thickness in the maxilla at the site of 1–1 at 6 mm level in class I patients, at the site of 5–6 at 2 mm level in class II patients, and at the site of 5–6 at 4 mm level in class III patients. In the entire maxilla, the maximum thickness was noted at the site of 1–1 at 6 mm level in class I patients. Al-Amri et al. [11] Baumgaertel and Hans [18], and Hu et al. [33] showed the maximum buccal cortical plate thickness in the maxilla at the sites of 5–6 and 6–7. This thickness increased towards the incisors. Khumsarn et al. [24] reported the maximum buccal cortical thickness at the site of 6–7 at 10 mm level in class I patients and at the site of 4–5 in class II patients, similar to the study by Fayed et al. [27]. Lim et al. [14] reported the maximum buccal cortical thickness at the sites of 2–3 and 3–4.

The palatal cortical plate was thin in the anterior region (1–1) in the present study. Its thickness increased at the site of 3–4 and then decreased. Al-Amri et al. [11] also reported that the palatal plate thickness decreased from the anterior towards the posterior region while Hu et al. [33] indicated slightly thicker palatal cortical plate in the posterior region, compared with the anterior region. This difference may be due to different measurement techniques since they measured the values on CT scans of the skull while we made the measurements on CBCT scans of patients. In the present study, the maximum palatal cortical plate thickness was noted at the site of 3–4 at 6 mm level in class I, at the site of 1–2 at 6 mm level in class II, and at the site of 2–3 at 6 mm level in class III patients. These results were in line with those of Fayed et al. [27].

The present results indicated that in all three classes, the buccal cortical plate thickness of the mandible was maximum at the site of 6–7. In line with the present results, Hu et al. [33] and Lim et al. [14] demonstrated increasing thickness of buccal cortical plate from the anterior towards the posterior region. Khumsarn et al. [24] Fayed et al. [27] and Park and Cho [32] also reported results similar to our findings. Nucera et al. [8] reported that the mandibular buccal cortical plate thickness was adequate at the second molar site for placement of miniscrews.

In the present study, the maximum lingual cortical plate thickness was noted at the site of 3–4 at 6 mm distance in classes I and III and at the site of 3–4 at 4 mm distance in class II patients, which was in agreement with the results of Fayed et al. [27].

According to the present results, the maximum cancellous bone thickness in the maxilla and mandible was recorded at the site of 6–7 at 6 mm distance in all three classes. Similarly, Coşkun and Kaya [28] demonstrated greater cancellous bone thickness between the maxillary molars and at all interradicular distances in the mandible in class II patients.

The maximum alveolar process thickness in the maxilla and mandible was recorded at the site of 6–7 at 6 mm distance in all three classes. In agreement with the present results, Khumsarn et al. [24] (in both classes I and II), Fayed et al. [27] and Poggio et al. [30] reported maximum alveolar process thickness at the site of 6–7 in both jaws.

The maximum distance between the maxillary sinus floor and CEJ was noted at the site of 3–4 in all three classes. Also, this distance had a significantly decreasing trend from the site of 3–4 towards the posterior region. The minimum distance was recorded at the site of 6–7 in classes I and III and at the site of 5–6 in class II patients. Similarly, Al-Amri et al. [11] reported that this distance was greater in the anterior region and significantly decreased towards the posterior area. Also, Yang et al. [26] reported results similar to ours.

Considering all the above, determination of an ideal site for miniscrew placement varies among different individuals, and the decision in this regard should be made based on the anatomical parameters of each patient. However, it may be stated that the safest area for miniscrew placement in the mandible of all three classes is between the first and second molar teeth, but it is difficult to determine the safest place in the maxilla.

Assessment of skeletal parameters alone was a limitation of this study because soft tissue parameters are also important in selection of the most appropriate site for miniscrew insertion. Small sample size was another limitation of this study. Also, since the number of female patients was much higher than males, it was not possible to recruit equal number of males and females. Thus, comparison of males and females in this respect was not possible. However, the three groups were standardized regarding the male/female ratio. Furthermore, due to small sample size, presence of metal restorations was not considered as an exclusion criterion, which can also be responsible for variations in the results.

Further studies are required to assess the effect of skeletal parameters on the success rate of miniscrew insertion in orthodontic patients. Also, the effect of soft tissue parameters on the success rate of miniscrew placement should be investigated in future studies. Finally, a systematic review is required to reach a definite conclusion on this topic.

## Conclusion

The area with maximum interradicular distance and optimal alveolar bone thickness for miniscrew insertion varies in different individuals depending on their sagittal skeletal pattern. Thus, the decision in this respect should be made based on individual parameters of each patient.

## Data Availability

The data used to support the findings of this study were supplied by corresponding author under license and data will be available on request. Requests for access to these data should be made to corresponding author.

## Abbreviations

CBCT: Cone-beam computed tomography
CEJ: Cementoenamel junction

## References

[1] Pan F, Kau CH, Zhou H, Souccar N (2013). The anatomical evaluation of the dental arches using cone beam computed tomography–an investigation of the availability of bone for placement of mini-screws. Head Face Med.

[2] Moslemzadeh SH, Sohrabi A, Rafighi A, Kananizadeh Y, Nourizadeh A (2017). Evaluation of interdental spaces of the mandibular posterior area for orthodontic mini-implants with cone-beam computed tomography. J Clin Diagn Res.

[3] Kuroda S, Sugawara Y, Deguchi T, Kyung HM, Takano-Yamamoto T (2007). Clinical use of miniscrew implants as orthodontic anchorage: success rates and postoperative discomfort. Am J Orthod Dentofacial Orthop.

[4] Schätzle M, Männchen R, Zwahlen M, Lang NP (2009). Survival and failure rates of orthodontic temporary anchorage devices: a systematic review. Clin Oral Implants Res.

[5] Veli I, Uysal T, Baysal A, Karadede I (2014). Buccal cortical bone thickness at miniscrew placement sites in patients with different vertical skeletal patterns. J Orofac Orthop.

[6] Zhao H, Gu XM, Liu HC, Wang ZW, Xun CL (2013). Measurement of cortical bone thickness in adults by cone-beam computerized tomography for orthodontic miniscrews placement. J Huazhong Univ Sci Technolog Med Sci.

[7] Dharmadeep G, Naik MK, Reddy YM, Cheruluri S, Praveen Raj K, Reddy BR (2020). Three-dimensional evaluation of interradicular areas and cortical bone thickness for orthodontic miniscrew implant placement using cone-beam computed tomography. J Pharm Bioallied Sci.

[8] Nucera R, Lo Giudice A, Bellocchio AM, Spinuzza P, Caprioglio A, Perillo L (2017). Bone and cortical bone thickness of mandibular buccal shelf for mini-screw insertion in adults. Angle Orthod.

[9] Baumgaertel S (2011). Cortical bone thickness and bone depth of the posterior palatal alveolar process for mini-implant insertion in adults. Am J Orthod Dentofacial Orthop.

[10] Chang C, Liu SS, Roberts WE (2015). Primary failure rate for 1680 extra-alveolar mandibular buccal shelf mini-screws placed in movable mucosa or attached gingiva. Angle Orthod.

[11] Al Amri MS, Sabban HM, Alsaggaf DH, Alsulaimani FF, Al-Turki GA, Al-Zahrani MS (2020). Anatomical consideration for optimal position of orthodontic miniscrews in the maxilla: a CBCT appraisal. Ann Saudi Med.

[12] Marquezan M, Lima I, Lopes RT, Sant'Anna EF, de Souza MM (2014). Is trabecular bone related to primary stability of miniscrews?. Angle Orthod.

[13] Carrillo R, Rossouw PE, Franco PF, Opperman LA, Buschang PH (2007). Intrusion of multiradicular teeth and related root resorption with mini-screw implant anchorage: a radiographic evaluation. Am J Orthod Dentofacial Orthop.

[14] Lim JE, Lee SJ, Kim YJ, Lim WH, Chun YS (2009). Comparison of cortical bone thickness and root proximity at maxillary and mandibular interradicular sites for orthodontic mini-implant placement. Orthod Craniofac Res.

[15] Farnsworth D, Rossouw PE, Ceen RF, Buschang PH (2011). Cortical bone thickness at common miniscrew implant placement sites. Am J Orthod Dentofacial Orthop.

[16] Samrit V, Kharbanda OP, Duggal R, Seith A, Malhotra V (2012). Bone density and miniscrew stability in orthodontic patients. Aust Orthod J.

[17] Jung YR, Kim SC, Kang KH, Cho JH, Lee EH, Chang NY (2013). Placement angle effects on the success rate of orthodontic microimplants and other factors with cone-beam computed tomography. Am J Orthod Dentofacial Orthop.

[18] Baumgaertel S, Hans MG (2009). Buccal cortical bone thickness for mini-implant placement. Am J Orthod Dentofacial Orthop.

[19] Mohammed H, Wafaie K, Rizk MZ, Almuzian M, Sosly R, Bearn DR (2018). Role of anatomical sites and correlated risk factors on the survival of orthodontic miniscrew implants: a systematic review and meta-analysis. Prog Orthod.

[20] Araghbidikashani M, Golshah A, Nikkerdar N, Rezaei M (2016). In-vitro impact of insertion angle on primary stability of miniscrews. Am J Orthod Dentofacial Orthop.

[21] Miyawaki S, Koyama I, Inoue M, Mishima K, Sugahara T, Takano-Yamamoto T (2003). Factors associated with the stability of titanium screws placed in the posterior region for orthodontic anchorage. Am J Orthod Dentofacial Orthop.

[22] Masumoto T, Hayashi I, Kawamura A, Tanaka K, Kasai K (2001). Relationships among facial type, buccolingual molar inclination, and cortical bone thickness of the mandible. Eur J Orthod.

[23] Horner KA, Behrents RG, Kim KB, Buschang PH (2012). Cortical bone and ridge thickness of hyperdivergent and hypodivergent adults. Am J Orthod Dentofacial Orthop.

[24] Khumsarn N, Patanaporn V, Janhom A, Jotikasthira D (2016). Comparison of interradicular distances and cortical bone thickness in Thai patients with Class I and Class II skeletal patterns using cone-beam computed tomography. Imaging Sci Dent.

[25] Al-Masri MM, Ajaj MA, Hajeer MY, Al-Eed MS (2015). Evaluation of bone thickness and density in the lower incisors' region in adults with different types of skeletal malocclusion using cone-beam computed tomography. J Contemp Dent Pract.

[26] Yang L, Li F, Cao M, Chen H, Wang X, Chen X (2015). Quantitative evaluation of maxillary interradicular bone with cone-beam computed tomography for bicortical placement of orthodontic mini-implants. Am J Orthod Dentofacial Orthop.

[27] Fayed MM, Pazera P, Katsaros C (2010). Optimal sites for orthodontic mini-implant placement assessed by cone beam computed tomography. Angle Orthod.

[28] Coşkun İ, Kaya B (2019). Relationship between alveolar bone thickness, tooth root morphology, and sagittal skeletal pattern : A cone beam computed tomography study. J Orofac Orthop.

[29] Ludwig B, Glasl B, Bowman SJ, Wilmes B, Kinzinger GS, Lisson JA (2011). Anatomical guidelines for miniscrew insertion: palatal sites. J Clin Orthod.

[30] Poggio PM, Incorvati C, Velo S, Carano A (2006). "Safe zones": a guide for miniscrew positioning in the maxillary and mandibular arch. Angle Orthod.

[31] Chaimanee P, Suzuki B, Suzuki EY (2011). "Safe zones" for miniscrew implant placement in different dentoskeletal patterns. Angle Orthod.

[32] Park J, Cho HJ (2009). Three-dimensional evaluation of interradicular spaces and cortical bone thickness for the placement and initial stability of microimplants in adults. Am J Orthod Dentofacial Orthop.

[33] Hu KS, Kang MK, Kim TW, Kim KH, Kim HJ (2009). Relationships between dental roots and surrounding tissues for orthodontic miniscrew installation. Angle Orthod.

[34] Moslemzade SH, Kananizadeh Y, Nourizadeh A, Sohrabi A, Panjnoosh M, Shafiee E (2014). Evaluation of cortical bone thickness of mandible with cone beam computed tomography for orthodontic mini implant installation. ABCmed.

[35] Lee KJ, Joo E, Kim KD, Lee JS, Park YC, Yu HS (2009). Computed tomographic analysis of tooth-bearing alveolar bone for orthodontic miniscrew placement. Am J Orthod Dentofacial Orthop.

[36] Motoyoshi M (2011). Clinical indices for orthodontic mini-implants. J Oral Sci.

